# The effectiveness of differential learning in small-sided soccer games for skill development in U20 amateur male players

**DOI:** 10.5114/biolsport.2025.151661

**Published:** 2025-08-29

**Authors:** Jaouher Hamaidi, Wissem Dhahbi, Mohamed Mansour Bouzourraa, Noomen Guelmami, Mohamed Ben Aissa, Wassim Moalla, Ismail Dergaa, Katja Weiss, Thomas Rosemann, Beat Knechtle, Makrem Zghibi

**Affiliations:** 1 High Institute of Sport and Physical Education of Ksar-Said, University of Manouba, Manouba 2010, Tunisia; 2 Department of Human and Social Sciences, High Institute of Sport and Physical Education of Kef, University of Jendouba, Kef 7100, Tunisia; 3 Research Unit “Sport Sciences, Health and Movement”, Higher Institute of Sports and Physical Education of Kef, University of Jendouba, Kef, Tunisia; 4 Training Department, Police College, Qatar Police Academy, Doha, Qatar; 5 Research Unit Physical Activity, Sport, and Health, UR18JS01, National Observatory of Sport, Tunis, Tunisia; 6 LR 19JS01 EM2S, Education, Motricity, Sport and Health, High Institute of Sport and Physical Education, University of Sfax, Tunisia; 7 Primary Health Care Corporation (PHCC), Doha, Qatar; 8 Institute of Primary Care, University of Zurich, Zurich, Switzerland; 9 Medbase St.Gallen am Vadianplatz, St. Gallen, Switzerland

**Keywords:** Adaptive learning, Football, Motor skill learning, Player development, Performance metrics

## Abstract

Traditional soccer training relies on repetitive drills, while modern approaches emphasize personalized strategies that better suit individual player development needs. This study examined the effects of Small-Sided Games (SSGs) alone and in combination with Differential Learning (SSG + DL) on physiological responses, tactical behaviors, and technical skills enhancement of soccer players. Twenty male soccer players participated in this randomized controlled trial, assigned to either a control group (SSG, n = 10, age: 19.4 ± 1.07 years) or an intervention group (SSG + DL, n = 10, age: 18.2 ± 0.91 years). Physiological responses (heart rate, blood lactate, RPE), tactical behaviors, and technical skills were assessed before and after an 8-week training program (four sessions weekly). Large main effects of Time were found for technical/tactical skills including possession (η^2^ = 0.83), passes (η^2^ = 0.86), shots (η^2^ = 0.77), tackles (η^2^ = 0.73), and decisionmaking (η^2^ = 0.92). Medium effects emerged for heart rate (η^2^ = 0.19) and effort (η^2^ = 0.27). Group effects were negligible for physical measures. Significant interaction effects favoring the intervention were found for possession (η^2^ = 0.42), passes (η^2^ = 0.42), tackling (η^2^ = 0.74), and marking (η^2^ = 0.58). The intervention group showed larger improvement effect sizes (g = 0.11–2.61) compared to controls (g = 0.05–1.97). Integration of SSGs with DL significantly enhances tactical behaviors and technical skills in amateur soccer players compared to SSGs alone. These findings provide coaches with a practical framework to develop more adaptable players, particularly valuable for youth teams with limited resources, highlighting the importance of incorporating innovative training methods that emphasize variability and exploration.

## INTRODUCTION

Effective management of training and learning methods is crucial for success in soccer skill development [1–3]. Researchers are continuously exploring innovative methodologies to enhance performance proficiency and evaluate different approaches [3]. The structure of training sessions and handling variations during practice play a significant role in fostering physical abilities, technical skills, and creative behavior [4, 5].

Traditional approaches, which relied on extensive repetition of optimal movements, have been challenged in the last decade. Scientists challenged conventional approaches, exploring personalized movement patterns and the rarity of identical movements occurring within and across individuals [6]. Among these, differential learning (DL) and small-sided games (SSGs) have gained considerable attention [7–9].

DL and SSGs, both dynamic and nonlinear methods, employ variability during practice to replicate the movement demands and technical requirements essential for skill learning [10, 11]. Recent studies have further expanded our understanding of the learning benefits associated with differential approaches [12]. Chow et al. [7] demonstrated that nonlinear pedagogy effectively facilitates game skill acquisition in territorial games, emphasizing the importance of variability in practice design [7]. Additionally, Rico-González et al. [11] highlighted that differential learning approaches particularly enhance creative and tactical behavior in soccer, providing coaches with practical applications for designing training tasks [13]. Deuker et al. [9] advocated for training methodologies that closely resemble game conditions, supporting the “train as you play” philosophy to improve effectiveness in youth soccer players [18]. Anwar et al. [14] found that eight weeks of small-sided games significantly enhanced passing accuracy and eye-foot coordination in soccer players, demonstrating the effectiveness of SSGs for specific skill development. The integration of both approaches may provide complementary benefits, addressing limitations identified in isolated applications of either method. They offer learners a variable environment to enhance decisionmaking skills and situational awareness, prioritizing personalized training that targets individual needs [15]. Both methods foster motivation, creativity, problem-solving, and cognitive flexibility among learners, providing a comprehensive approach to skill development and performance optimization [15, 16]. The DL model, which was developed in the late [17], suggests that successful learning requires introducing variations by integrating unpredictable events [6]. These variations are adjusted according to the learner’s individual situational attributes throughout the learning process [18], creating an environment that increases the number of movement variations by avoiding repetition and correction during any skill acquisition [19]. It is a motor learning method that integrates substantial variability during practice sessions, helping subjects to find performance models during complex motor skills situations [20, 21]

SSGs, on the other hand, are a pedagogical approach employed to enhance learners’ performance. SSGs are variations of regular soccer matches played on smaller pitch areas with modified rules and fewer players [22]. This approach divides players into reduced groups, often with modified rules or playing conditions designed to accentuate specific skills or tactical objectives [23]. SSGs, augmented by various rule adjustments, have been recommended as an effective strategy for exposing players to scenarios similar to those encountered in competitive matches [24]. Most of the research on SSGs has focused on their application as a training technique, highlighting their ability to replicate the movement patterns, physical demands, and technical skills required in real competitive matches [22]. SSGs replicate the movement patterns, physical demands, and technical skills required in real competitive matches, improving players’ decision-making and execution of technical-tactical actions [25].

While previous research has investigated the effects of SSGs and DL interventions on technical skill enhancement, there is a significant gap in the literature regarding direct comparisons between these two approaches [11, 25–27]. Thus, this study aimed to compare the impacts of SSGs per se and SSGs integrated with DL on the physiological responses, tactical behaviors, and technical skills enhancement of soccer players.

Despite emerging evidence supporting the efficacy of both SSGs and DL independently, there is limited research directly comparing their relative effectiveness when used in combination versus SSGs alone. This study aimed to address this research gap by investigating the following research questions: (1) How does the integration of DL with SSGs affect physiological responses in soccer players compared to SSGs alone? (2) Does combining DL with SSGs enhance tactical behaviors and decision-making capabilities beyond what can be achieved with SSGs alone? (3) To what extent does the integration of DL with SSGs improve technical skill execution compared to traditional SSG approaches?

Based on the established benefits of both DL and SSGs in soccer skill development, we hypothesized that the integration of DL into SSGs will result in significant improvements in both tactical decisionmaking and technical execution compared to SSGs alone. Specifically, we anticipated that the variability and unpredictability inherent in DL, when combined with the realistic match scenarios provided by SSGs, will create a training environment that accelerates skill acquisition, improves decision-making, and enhances overall performance.

## MATERIALS AND METHODS

### Ethical Approval

Ethical approval was obtained from the local Institutional Research Ethics (code: ph-0071-2023) and was conducted in accordance with the ethical principles outlined in the Declaration of Helsinki. All participants and their legal parents were informed of their rights, including the right to withdraw from the study at any time. Written informed consent was obtained from both the participants and their legal parents. It also complied with the ethical and procedural requirements for the conduct of sports medicine and exercise science research [28].

### Participants

The study involved 20 male soccer players from the Amateur League. While our power analysis indicated that a sample size of 20 participants would exceed our power threshold (yielding an actual power of 0.84), we acknowledge that this relatively modest sample size may limit the generalizability of our findings. However, our sample size aligns with recent comparable studies in soccer skill development, such as those by Orangi et al. [4] (n = 24) and da Silva [29] (n = 18), which demonstrated significant effects with similar participant numbers. Participants were randomly and equally assigned into two groups of 10 using a computer-generated random number sequence (Random.org). This randomization was stratified by playing position to ensure balanced distribution of defenders, midfielders, and forwards across groups. The resulting groups were: SSG + DL (i.e., SSG with DL; age: 18.2 ± 0.91 years, body-mass: 65.5 ± 2.5 Kg and body height: 173.9 ± 2.60 cm) and SSGs (i.e., SSG without DL; age: 19.4 ± 1.07 years, body mass: 64.3 ± 2.02 Kg and body height: 174.7 ± 2.75 cm). Four goalkeepers were involved in the research; however, their data were omitted from the analysis due to their distinct and specialized role, which differs significantly from that of outfield players.

Prior to the experiment design, we conducted the power analysis for the planned ANOVA with repeated measures (between withinsubject and between-subject). We specified our inputs based on a medium effect size (*f* = 0.35), with an alpha error probability set at 0.05 and a power goal of 0.8. The analysis outputs indicated that a sample size of 20 participants would exceed our power threshold, yielding an actual power of 0.84. This suggests an enhanced likelihood of detecting the hypothesized effect, should it exist. The noncentrality parameter λ was calculated to be 9.8, while the critical Fvalue reached 4.42 with associated degrees of freedom allocated as 1 for the numerator and 18 for the denominator.

### Study design

The study was conducted from January 15, 2023, until March 15, 2023. A longitudinal repeated cross-sectional study design was used, involving repeated observations over an eight-week period. This study investigated the effectiveness of two different intensities (SSG + LD and SSGs) of simulated SSG soccer training program on subsequent physiological responses (e.g., Heart rate monitoring – HR, Blood Lactate concentration – [La], the Borg Rating of Perceived Exertion – RPE), tactical behaviors (e.g., game performance evaluation tool – G-PET, decision-making) and technical skills (e.g., possession, unsuccessful passing, successful passing, successful dribbling, unsuccessful shooting, successful shooting, unsuccessful tackling, interception, lost ball, control error). All sessions were performed at the same time of the day (i.e., between 17 h and 19 h) to minimize the effects of diurnal variations in the measured variables.

**TABLE 1:** Characteristics of SSG and SSG + DL groups

Minute	SSGs	SSG + DL	

	Without any instruction	Individual instructions	Collective instructions
**0–1’**	(1:2:3:1) formation without any variations	Pass only with right foot Pass using the inside of the foot arms crossed intercept the ball without any physical contact with adverse player	(1:3:2:1) achieve five passes before shooting

**1–2’**	(1:2:3:1) formation ithout any variations	Pass only with left foot pass using the outside of the foot arms crossed intercept the ball without any physical contact with adverse player	(1:2:3:1) achieve five passes before shooting

**2–3’**	(1:2:3:1) formation without any variations	Pass the ball, alternating between using the inside and outside of the foot Hands on the head intercept the ball without any physical contact with adverse player	(1:1:2:3) achieve five passes before shooting

SSG = small sided games, DL = differential learning.

### Procedures

Before data collection, participants were oriented and familiarized with the Small-Sided Games (SSG) and SSG + DL procedures, including the associated equipment. Anthropometric measurements, such as body mass and height, were recorded prior to the training session according to Kushner’s protocol. The inclusion of physiological measures (HR, blood lactate, and RPE) in this study served multiple purposes [30]. First, these parameters provided objective quantification of internal training load during both intervention protocols, ensuring comparable exertion levels between groups despite different methodological approaches. Second, previous research by Frank et al. [21] has demonstrated that DL approaches can influence physiological responses differently than traditional training methods, potentially affecting neural activation patterns and subsequent skill acquisition processes. Third, understanding the relationship between physiological stress and motor learning outcomes is critical when implementing novel training methodologies in practical settings. As noted by Clemente et al. [31], the fatigue induced by training interventions can significantly impact technical performance and learning consolidation. Thus, monitoring physiological responses allowed us to examine whether the integration of DL affected the physiological demands of SSGs, which could potentially influence the observed learning outcomes. Body mass was assessed using a calibrated digital scale (SECA Model 861, Vogel and Halke, Hamburg, Germany), while height was measured using a wall-mounted stadiometer.

All participants performed a standardized warm-up protocol before soccer training sessions. This 10-minute program began with a 2-minute light jog, followed by 5 minutes of dynamic stretching exercises, and concluded with 3 minutes of technical ball drills.

The training involved SSG and SSG + DL in a 5 v 5 format with 2 goalkeepers on a 40 × 30 m pitch. Both training interventions (SSG and SSG + DL) were precisely time-matched to ensure equitable training exposure. Each group participated in identical session durations (90 minutes per session), training frequency (four sessions per week), and total intervention period (eight weeks). Within each 90-minute session, both groups completed the same number of game repetitions (four 3-minute bouts with 1-minute rest intervals) and identical work-to-rest ratios. The only systematic difference between conditions was the introduction of differential learning elements in the SSG + DL group, while the SSG group performed the same smallsided games without these variations. This standardization of training volume and duration is critical for isolating the effects of the differential learning approach from potential confounding variables related to training load or exposure. Each session consisted of four 3-minute bouts with 1-minute rest intervals. Post-training assessments included physiological responses such as maximal heart rate (HRmax), RPE, and [La] levels. Technical skills and tactical behaviors were evaluated using a video recorder (Sony HD Video Recording HD RC X 405 camera) and the Game Performance Evaluation Tool (GPE-T), respectively.

The training program was conducted four times weekly for eight weeks from 17:00 to 18:30 h PM on an artificial turf field with temperatures between 20°C and 25°C. Post-intervention assessments were conducted to evaluate changes and improvements in physiological responses, technical skills, and tactical behaviors.

**TABLE 2:** Description of Decision-Making measurement using the Game Performance Evaluation Tool (G-PET)

Level 1: Selection of Technical Skills	Level 2: Decision-Making Adaptation of Tactical context
**Defender with ball:** Marking (with ball) Shot Blocked Entry Defensive aids (with ball)	Assessing a defender’s decision-making and tactical awareness with the ball involves evaluating their situational awareness, tactical adaptability, and execution of actions. Key criteria include awareness of teammates and opponents, tactical understanding, anticipation skills, and execution quality. Defender’s risk management, alignment with team objectives, and contribution to team play should be considered too.

**Defender without ball:** Marking (without ball) Interception Defensive aids (without ball)	Effective defenders without the ball demonstrate the ability to maintain proper positioning relative to the ball, opponents, and teammates, allowing them to anticipate and intercept passes or block shots effectively. Additionally, they reveal strong communication skills by providing clear instructions to teammates and maintaining awareness of their surroundings. Decision-making plays an important role as defenders without the ball must quickly assess the situation and choose the most appropriate course of action, such as applying pressure to the opponent, covering passing lanes, or supporting teammates.

**Striker with Ball:** Passing Shooting Driving	Evaluating a striker with the ball involves assessing several key criteria to gauge their performance and decision-making abilities. Firstly, their ability to create goal-scoring opportunities by exploiting defensive weaknesses and finding space in the attacking third is essential. This includes their proficiency in dribbling, shooting, and passing accurately to teammates in advantageous positions. Additionally, their awareness of the tactical context, such as the positioning of defenders and the goalkeeper, and their ability to adapt their actions accordingly is crucial. A striker’s decisionmaking, especially in difficult situations such as one-on-one encounters with the goalkeeper or tight defensive formations, reflects their composure and strategic thinking.

**Striker without ball**	Assessing a striker’s performance without the ball involves evaluating their movement and positioning off-the-ball to create scoring opportunities for themselves and teammates. This assessment includes their ability to anticipate plays, read the game, and execute well-timed runs into space to receive passes. Effective positioning relative to the opposition’s defensive line is crucial, as is the capacity to exploit defensive gaps. Additionally, the striker’s work rate and their contribution to pressing and pressuring opponents when the team is not in possession should be measured.

### Physiological responses

– *Heart Rate (HR):* HR was monitored using the Polar Team Sport System (Polar-Electro OY, Kempele, Finland).
– *Blood Lactate Concentration ([La]):* [La] levels (mmol · L^−1^) were determined by analyzing capillary blood samples from the earlobe using a handheld analyzer (Lactate Scout, EKF, SencLab, Magdeburg, Germany). Samples were collected three minutes post-testing session.
– *Borg Rating of Perceived Exertion (RPE):* The Borg CR10 scale, a category-ratio (CR) scale ranging from 0 to 10, was utilized to assess perceived effort during exercise.

### Tactical behaviors

– *Game Performance Evaluation Tool (G-PET):* G-PET was developed by French & Thomas [32] and further refined by Nevett et al., [33] was utilized to analyze game performance components and development characteristics. The tool’s reliability was confirmed through testing, with correlation coefficients exceeding 0.80 and inter-observer correlations for all categories ranging from 0.77 to 1.00 [34].
  The G-PET evaluated decision-making at two levels, with each skill’s success or failure recorded as either (1) or (0). The first level assessed decision-making in relation to skill or movement execution. For instance, a correct decision (1) would be passing the ball to an unmarked teammate, while an incorrect decision (0) would be moving into a space occupied by an opponent. The second level analyzed players’ adaptability to tactical situations by evaluating their tactical intention in each action.

### Technical skills

The study assessed various technical skills relevant to football performance. These included:

– *Possession:* The duration a player maintains control of the ball.
– *Passing:* Successful passes made by the player and unsuccessful or intercepted passes.
– *Dribbling:* Successful dribbling maneuvers executed by the player and unsuccessful or intercepted attempts to advance with the ball.
– *Shooting:* Successful goal-scoring attempts and unsuccessful attempts to score.
– *Tackling:* Successful defensive challenges executed by the player and unsuccessful or mistimed defensive challenges.
– *Interception:* Instances where the player successfully intercepts an opponent’s pass or clearance.
– *Lost Ball:* Instances where the player loses possession due to a mistake or challenge from an opponent.
– *Control Error:* Instances where the player fails to control the ball effectively, leading to a loss of possession.

This comprehensive approach, aligned with previous research on football match analysis metrics [35], provides valuable insights into players’ technical proficiency and decision-making during training sessions.

### Statistical analysis

Data were summarized as mean and the standard deviation (± SD). Data were analyzed using SPSS for Windows (version 29, Inc., Chicago, IL, USA). After testing for normal distribution (Kolmogorov– Smirnov test), differences within and between groups were analyzed using a two-factors mixed ANOVA (2 × 2). The within-subjects factor is Time (pre- and post-test), with the between-subjects factor being Group (SSG + LD and SSGs). Greenhouse-Geisser corrections were used when the assumption of sphericity (Mauchly’s test) was violated. To help protect against type II errors, an estimated effect size was presented using partial eta squared η^2^. η^2^ was interpreted as small (0.01), medium (0.06), and large (0.14).

After confirming significant factors interactions, a Bonferroni-adjusted pairwise post-hoc test was performed. Effect sizes for pairwise comparisons were calculated as Hedges’ g, while comparisons between the percentage of changes in the group were performed by one-way ANOVA. A Hedges’ g was categorized as 0.2 = small effect, 0.5 = moderate effect, and 0.8 = large effect and a p < 0.05 was accepted as the minimal level of statistical significance.

## RESULTS

### Effects of Time, group and interaction (group*time)

**FIG. 1:**
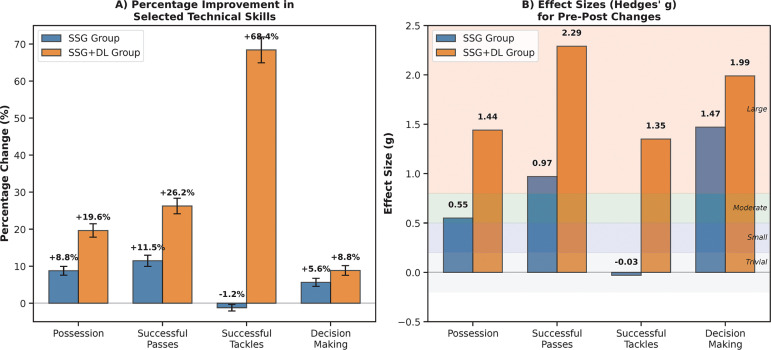
Effects of small-sided games (SSG) and small-sided games with differential learning (SSG + DL) on performance metrics in under-20 amateur soccer players Panel A displays the percentage improvement from pre-test to post-test for both training groups across four critical performance indicators: possession maintenance, successful passing execution, successful tackling, and decision-making capacity. Error bars represent standard error of the mean. Panel B presents the corresponding effect sizes (Hedges’ g) for each metric, with values interpreted as trivial (0–0.2), small (0.2–0.5), moderate (0.5–0.8), and large (> 0.8). The SSG + DL group demonstrated consistently greater improvements across all metrics, with particularly notable enhancements in successful tackling (68.41% improvement, g = 1.35) compared to the SSG-only group (-1.22% change, g = -0.03).

Results are organized by measurement category (physiological, technical, and tactical) and presented in Table 3, Table 4, and Figure 1. For physiological measures, we found medium main effects of Time for maximal heart rate (η^2^ = 0.19) and rating of perceived exertion (η^2^ = 0.27), while no significant effects were observed for lactate concentration. For technical skills, large main effects of Time were found for possession (η^2^ = 0.83), successful passes (η^2^ = 0.86), successful shots (η^2^ = 0.77), successful tackles (η^2^ = 0.73), control errors (η^2^ = 0.36), and lost balls (η^2^ = 0.60). For tactical behavior, large main effects of Time were observed for defender with/without ball (η^2^ range 0.62–0.73) and decision making (η^2^ = 0.92).

**TABLE 3:** Results of ANOVA with 2 × 2 repeated measures

	Main Effect								Interaction effect			

	Time				Group				Time × Group			

	F	Sig.	*η^2^*	Decision	F	Sig.	*η^2^*		F	Sig.	*η^2^*	Decision
Hrmax ()	4.13	0.057	0.19	(Medium)	0.00	0.961	0.00	(Small)	1.06	0.317	0.06	(Small)
RPE	6.67	0.019	0.27	(Medium)	0.37	0.550	0.02	(Small)	0.14	0.712	0.01	(Small)
Lactate	0.12	0.738	0.01	(Medium)	2.17	0.158	0.11	(Small)	0.00	0.992	0.00	(Small)
Possession	89.34	0.000	0.83	(Large)	0.29	0.599	0.02	(Small)	12.78	0.002	0.42	(Large)
Unsuc-Pass	17.51	0.001	0.49	(Large)	3.08	0.096	0.15	(Small)	9.65	0.006	0.35	(Small)
Suc-Pass	114.72	0.000	0.86	(Large)	2.39	0.140	0.12	(Small)	12.99	0.002	0.42	(Large)
Unsuc-Drib	57.68	0.000	0.76	(Large)	3.14	0.093	0.15	(Small)	8.26	0.010	0.31	(Small)
Suc-Drib	4.12	0.058	0.19	(Medium)	1.80	0.196	0.09	(Small)	0.01	0.930	0.00	(Small)
Unsuc-Shoot	0.78	0.387	0.04	(Medium)	0.11	0.739	0.01	(Small)	0.74	0.401	0.04	(Small)
Suc-Shoot	59.42	0.000	0.77	(Large)	0.07	0.790	0.00	(Small)	0.84	0.370	0.04	(Small)
Unsuc-Tack	1.57	0.226	0.08	(Medium)	1.48	0.240	0.08	(Small)	1.55	0.229	0.08	(Small)
Suc-Tack	47.73	0.000	0.73	(Large)	0.00	0.959	0.00	(Small)	49.94	0.000	0.74	(Large)
ControlErr	10.04	0.005	0.36	(Large)	0.03	0.863	0.00	(Small)	0.47	0.501	0.03	(Small)
Intercept	0.36	0.559	0.02	(Medium)	2.37	0.141	0.12	(Small)	0.63	0.437	0.03	(Small)
LostBall	26.71	0.000	0.60	(Large)	0.57	0.459	0.03	(Small)	0.16	0.692	0.01	(Small)
DWB	49.45	0.000	0.73	(Large)	0.91	0.352	0.05	(Small)	0.84	0.372	0.04	(Small)
DWOB	29.44	0.000	0.62	(Large)	0.09	0.763	0.01	(Small)	1.93	0.182	0.10	(Small)
SWOB	28.37	0.000	0.61	(Large)	0.31	0.585	0.02	(Small)	3.22	0.090	0.15	(Small)
SWB	29.31	0.000	0.62	(Large)	2.63	0.122	0.13	(Small)	2.27	0.149	0.11	(Small)
DM	201.12	0.000	0.92	(Large)	0.01	0.931	0.00	(Small)	25.10	0.000	0.58	(Large)

Hr_max_ = maximal heart rate, RPE = Rating of Perceived Exertion, Lactate = Blood Lactate Concentration, Unsuc-Pass = unsuccessful passes, Suc-Pass = successful passes, Unsuc-Drib = unsuccessful dribbles, Suc-Drib = successful dribbles, Unsuc-Shoot = unsuccessful shooting, Suc-Shoot = successful shooting, Unsuc-Tack = unsuccessful tackling, Suc-Tack = successful tackling, ControlErr = control errors, Intercept = interception, LostBall = lost ball, DWB = defender with ball, DWOB = defender without ball, SWOB = striker with ball, SWB = striker without ball, DM = decision making, η^2^ = Partial Eta Squared

**TABLE 4:** Comparative analysis of pre-post intervention performance metrics among groups

	Small-Sided Games (SSGs)							Small-Sided Games with Differential Learning (SSG+DL)						
	
	Pre		Post					Pre		Post				
	Mean	SD	Mean	SD	Change %	g	Decision	Mean	SD	Mean	SD	Change %	g	Decision
Hrmax (bpm)	83.06	2.25	84.90	2.43	2.21%	1.09	Large	83.65	1.73	84.25	2.17	0.72%	0.39	Large
RPE	7.08	1.17	7.90	0.84	11.48%	0.73	Large	7.36	0.68	7.97	0.82	8.25%	0.63	Large
Lactate (mmol/l)	3.56	0.59	3.59	0.61	0.90%	0.04	Small	3.86	0.46	3.90	0.30	0.88%	0.05	Large
Possession	9.41	1.52	10.23	2.12	8.75%	0.55	Large	9.29	1.37	11.11	1.25	19.64%	1.44	Large
Unsuc-Pass	2.36	0.28	2.25	0.54	-4.46%	-0.15	Small	2.39	0.33	1.68	0.42	-29.72%	-1.05	Large
Suc-Pass	10.15	1.32	11.31	1.02	11.46%	0.97	Large	10.32	1.05	13.03	1.24	26.25%	2.29	Large
Unsuc-Drib	3.30	0.22	3.07	0.23	-7.23%	-0.46	Small	3.13	0.52	2.53	0.37	-19.18%	-0.81	Large
Suc-Drib	3.14	0.34	3.45	0.40	9.80%	0.46	Medium	3.41	0.75	3.69	0.53	8.28%	0.32	Large
Unsuc-Shoot	2.74	0.47	2.74	0.30	-0.11%	0	Small	2.79	0.54	2.58	0.39	-7.52%	-0.28	Large
Suc-Shoot	4.41	0.53	4.86	0.65	10.12%	0.52	Large	4.51	0.32	4.87	0.40	7.78%	0.53	Large
Unsuc-Tack	1.36	0.41	1.04	0.50	-23.36%	-0.43	Small	1.31	0.24	1.31	0.16	-0.08%	0.00	Large
Suc-Tack	2.22	0.72	2.19	0.72	-1.22%	-0.03	Small	2.03	0.84	3.41	0.86	68.41%	1.35	Large
ControlErr	1.41	0.72	1.41	0.50	-0.07%	0	Small	1.10	0.30	1.03	0.31	-6.21%	-0.11	Large
Intercept	3.53	0.90	3.39	0.66	-3.77%	-0.14	Small	3.92	0.60	3.94	0.68	0.48%	0.02	Large
LostBall	2.05	0.43	2.02	0.40	-1.66%	-0.05	Small	1.76	0.49	1.49	0.47	-15.56%	-0.36	Large
DWB	66.93	3.18	70.85	3.87	5.85%	1.88	Large	67.79	3.15	72.97	4.15	7.66%	2.45	Large
DWOB	50.41	2.71	53.83	2.56	6.78%	1.9	Large	49.50	3.14	55.67	2.77	12.48%	3.24	Large
SWOB	56.24	3.54	59.65	3.47	6.06%	1.64	Large	56.12	4.50	62.18	4.31	10.81%	2.61	Large
SWB	63.48	2.92	65.88	2.50	3.78%	1.32	Large	64.88	3.94	69.13	4.29	6.55%	1.89	Large
DM	41.43	2.16	43.77	1.96	5.64%	1.47	Large	42.16	2.77	45.88	2.97	8.84%	1.99	Large

Hr_max_ = maximal heart rate, RPE = Rating of Perceived Exertion, Lactate = Blood Lactate Concentration, Unsuc-Pass = unsuccessful passes, Suc-Pass = successful passes, Unsuc-Drib = unsuccessful dribbles, Suc-Drib = successful dribbles, Unsuc-Shoot = unsuccessful shooting, Suc-Shoot = successful shooting, Unsuc-Tack = unsuccessful tackling, Suc-Tack = successful tackling, ControlErr = control errors, Intercept = interception, LostBall = lost ball, DWB = defender with ball, DWOB = defender without ball, SWOB = striker without ball, SWB = striker with ball, DM = decision making, SD = standard deviation

These large effect sizes demonstrate the intervention program significantly improved players’ technical skills, tactical abilities and decision making over time.

Medium main effects of Time were observed for maximal heart rate (η^2^ = 0.19), rating of perceived exertion (η^2^ = 0.27), and successful (η^2^ = 0.19) and unsuccessful dribbles (η^2^ = 0.76). The physiological load and selected technical skills were moderately enhanced during the training.

For the physical measures, small and non-significant main effects of Group were found for maximal heart rate (η^2^ = 0.00), rating of perceived exertion (η^2^ = 0.02), and lactate concentration (η^2^ = 0.11). While group means differed slightly for lactate at baseline, the intervention and control teams did not significantly vary in physical fitness prior to training.

Regarding technical and tactical performance, negligible main effects of Group were observed for possession (η^2^ = 0.02), successful tackles (η^2^ = 0.00), control errors (η^2^ = 0.00), defender/striker with/without ball (η^2^ range 0.00–0.05). This suggests the teams exhibited comparable skill in these areas at pre-testing.

Smaller yet still non-significant effects emerged for unsuccessful passes and dribbles (η^2^ = 0.15), successful passes/dribbles/shoots (η^2^ range 0.00–0.12), unsuccessful tackles/intercepts/ball losses (η^2^ range 0.01–0.08), and decision making (η^2^ = 0.00). While baseline scores varied slightly, the differences were not meaningful.

There were no other significant main effects of Time for unsuccessful passes/shots, unsuccessful tackles, or lactate concentration. While mean scores changed over time for some variables, the differences were not statistically significant. The 2 × 2 repeated measures ANOVAs revealed significant Time × Group interaction effects for possession (F_(1.23)_ = 12.78, p = 0.002, η^2^ = 0.42) and successful passes (F_(1.23)_ = 12.99, p = 0.002, η^2^ = 0.42), with large effect sizes. For both variables, the intervention group improved from pre- to post-test to a greater extent than the control group.

As illustrated in Figure 1, the percentage improvements were consistently greater in the SSG + DL group compared to the SSG group across key performance metrics. The practical significance of these statistical findings is particularly evident in the successful tackles parameter, where the SSG + DL group demonstrated a 68.41% improvement compared to a slight decline (-1.22%) in the SSG group. This suggests that the integration of differential learning principles substantially enhances defensive skill acquisition. Similarly, the superior improvement in decision-making (8.84% vs. 5.64%) indicates enhanced tactical awareness and game intelligence when differential learning is incorporated. These findings have immediate practical applications for coaches seeking to accelerate player development, particularly in defensive actions and tactical decision-making.

There were no significant interaction effects for HRmax (F_(1.23)_ = 1.06, p = 0.317, η^2^ = 0.06), RPE (F_(1.23)_ = 0.14, p = 0.712, η^2^ = 0.01), or lactate (F_(1.23)_ = 0.00, p = 0.992, η^2^ = 0.00), with small effect sizes observed. While mean differences existed between groups over time for some variables, the changes were not statistically significant.

There were no significant Time × Group interaction effects for successful dribbles (F_(1.23)_ = 0.01, p = 0.930, η^2^ = 0.00), unsuccessful shots (F_(1.23)_ = 0.74, p = 0.401, η^2^ = 0.04), successful shots (F_(1.23)_ = 0.84, p = 0.370, η^2^ = 0.04), unsuccessful tackles (F_(1.23)_ = 1.55, p = 0.229, η^2^ = 0.08), control errors (F_(1.23)_ = 0.47, p = 0.501, η^2^ = 0.03), intercepts (F_(1.23)_ = 0.63, p = 0.437, η^2^ = 0.03), lost balls (F_(1.23)_ = 0.16, p = 0.692, η^2^ = 0.01), defender with ball (F_(1.23)_ = 0.84, p = 0.372, η^2^ = 0.04), defender without ball (F_(1.23)_ = 1.93, p = 0.182, η^2^ = 0.10), or striker without ball (F_(1.23)_ = 3.22, p = 0.090, η^2^ = 0.15), showing small effect sizes.

However, there were large and significant interaction effects for successful tackles (F_(1.23)_ = 49.94, p = 0.000, η^2^ = 0.74) and decision making (F_(1.23)_ = 25.10, p = 0.000, η^2^ = 0.58). This suggests the intervention was especially effective at improving defensive skills, such as tackling from pre- to post-, compared to any changes in the control group over the same period.

### Post-Hoc tests Analysis

For pairwise comparisons, Successful Shots (Suc-Shoot) experienced an improvement (p < 0.001, g = 0.53, Large), and Successful Tackles (Suc-Tack) showed significant enhancement (p < 0.001, g = 1.35, Large). Control Error (ControlErr), on the other hand, saw a minimal yet significant change (p < 0.05, g = 0.11, Small), and Lost Balls (LostBall) indicated a moderate improvement (p < 0.01, g = 0.36, Large).

Defensive activities, measured as Defender with Ball (DWB) and Defender without Ball (DWOB), revealed very substantial enhancements (p < 0.001, g = 2.45 and g = 3.24, respectively, both with a rating of Large). Similar significant and large effects were observed for Striker without Ball (SWOB, p < 0.001, g = 2.61) and Striker with Ball (SWB, p < 0.001, g = 1.89). Lastly, Decision Making (DM) also showed a significant and sizeable improvement (p < 0.001, g = 1.99, Large).

These results suggest that the experimental intervention had a profound and statistically significant impact on the performance metrics of the experimental group, with most measures showing large effect sizes, reflecting meaningful improvements in the tested abilities.

In the control group, the Bonferroni post hoc analysis revealed substantial effects across several measures. Possession showed a significant effect with a sizeable impact on performance (p < 0.001, g = 0.55, Large). Successful Passing (Suc-Pass) exhibited one of the largest improvements (p < 0.001, g = 0.97, Large). Unsuccessful Dribbling (Unsuc-Drib) had a moderate yet significant effect (p < 0.01, g = 0.46, Medium), while Successful Shooting (Suc-Shoot) demonstrated a robust enhancement (p < 0.001, g = 0.52, Large). Changes in Lost Balls (LostBall) were significant but minimal (p < 0.01, g = 0.05, Small). Defender with Ball (DWB) and Defender without Ball (DWOB) showed very pronounced and significant effects (p < 0.001, g = 1.88; p < 0.05, g = 1.90, respectively, both with a rating of Large). Similarly, Striker without Ball (SWOB) and Striker with Ball (SWB) indicated significant, large improvements (p < 0.05, g = 1.64; p < 0.05, g = 1.32, respectively). Finally, Decision Making (DM) also registered a large and significant effect (p < 0.001, g = 1.47).

## DISCUSSION

This study investigated the effects of integrating differential learning (DL) into small-sided games (SSGs) on three outcomes: physiological responses, tactical behaviors and decision-making, and technical skill execution in soccer players. Overall, both groups experienced moderate improvements in maximal heart rate and perceived exertion over time, with no significant Time × Group interactions for these physiological measures, which indicate that DL did not differentially amplify cardiovascular load compared to SSGs alone. In contrast, the SSG + DL group demonstrated substantially greater gains in tactical awareness and defensive skill acquisition, particularly in decisionmaking and successful tackles, supported by large interaction effect sizes. Moreover, technical execution also benefited more from DL integration, as shown by significant Time × Group interactions for possession and successful passes, with post-hoc effect sizes ranging from moderate to very large across shots, dribbles, and positional tasks. Taken together, these findings suggest that adding DL to SSGs accelerates tactical and technical development beyond what traditional SSGs achieve, while eliciting similar physiological responses.

### Effects on Physiological Responses

Our analysis revealed that both the SSG-only and SSG + DL groups demonstrated moderate increases in maximal heart rate and RPE. Specifically, no significant Time × Group interactions were observed for heart rate, RPE or blood lactate. These findings are consistent with existing literature that suggests additional variability in smallsided games does not impose excessive cardiovascular demands.

In particular, Hill-Haas et al. [22] reported comparable heart-rate responses between traditional SSGs and those modified with variable constraints. Similarly, Gonçalves et al. [36] showed that restricting pitch areas reduced jogging and running intensities by 20–50% and 60–90%, respectively, and impaired overall distance covered and heart-rate responses. In contrast, player achieved substantial gains in tactical awareness and decision-making. These findings complement our own, suggesting that task constraints whether through spatial restrictions or the variability introduced by DL can be designed to affect tactical behavior rather than physiological load. Likewise, a study comparing different ball types in changed SSGs found that altering the ball type led to moderate decreases in total distance covered (11.1% and 6.2%, respectively) and jogging distance (11.9% and 8.0%), as well as reductions in most of the physical variables [37]. These findings underscore how variability can foster adaptive movement behaviors without compromising cardiovascular demands.

Furthermore, Ferrandis et al. [38] examined how changing tactical formation influenced locomotor and physiological loads during SSGs in youth players. While age, pitch size, and position each affected total distance, high-intensity running, speeds, and heart-rate responses (all p < 0.05), no significant effects of tactical formation emerged for any locomotor or physiological parameter. This suggests that varying tactical setups may not meaningfully alter the external or internal load in youth SSGs.

Most notably, Rampinini et al. [23] investigated how game format, pitch size, and coach encouragement influenced physiological responses in amateur players. They found significant main effects for all three factors. Importantly, no interactions emerged between exercise type, field dimensions, and encouragement (p > 0.15), indicating that each variable independently modulates internal load. Consequently, these findings confirm that the complex tasks of DL integrated into SSGs may enrich tactical and technical demands but do not significantly affect players’ physiological systems.

### Effects on Tactical Behaviors and Decision-Making

The enhanced performance outcomes in the SSG + DL group can be due to several factors, including variability, uncertainty, and nonlinearity during practice sessions. These factors, corroborated by previous studies [27, 39, 40], stimulate adaptive motor learning processes, augment perceptual-cognitive skills, and facilitate skill transfer to game situations [41]. The SSG + DL group, by participating in perpetually evolving and unpredictable training conditions, likely cultivated a more resilient and adaptable skill set, thereby enhancing their effectiveness in the dynamic and unpredictable nature of soccer [42]. This evidence highlights the efficacy of SSGs combined with DL in fostering technical skills and adaptability in soccer players.

The superior performance improvements observed in the SSG + DL group can be attributed to several underlying mechanisms. First, the intentional introduction of variability through DL likely stimulated neuroplastic adaptations in the central nervous system. According to Frank et al. [21], DL induces higher alpha and theta brainwave activity, which facilitates working memory processes and enhances motor learning consolidation. Second, the continuous exposure to novel movement challenges in the DL approach likely promoted greater cognitive flexibility, forcing players to constantly adapt their perceptual-cognitive processes when executing technical skills under varying constraints [43]. This cognitive engagement may strengthen the neural pathways associated with decision-making and motor control, leading to more robust skill acquisition [44]. Third, the unpredictable nature of DL exercises likely enhanced players’ attentional capabilities by requiring them to continuously anticipate and respond to changing task demands, thereby developing their ability to detect relevant environmental information more efficiently during gameplay [45]. Recent neuroimaging studies have demonstrated that variable practice conditions, similar to those employed in differential learning, lead to more extensive activation across motor cortical regions compared to repetitive practice [46], suggesting a broader neural representation of learned skills that may facilitate adaptability in dynamic game situations.

### Effects on Technical Skill Execution

In line with our findings, Schollhorn et al., [19] supported the superiority of the DL approach compared to traditional training approaches based on the results of soccer technique tests in their study. Similarly, Santos et al., [15] confirmed that the technical variability advocated by differential learning enhances the regularity of positioning behavior in soccer. Gaspar et al., [27] reported that an acute intervention employing DL methodologies induces noteworthy alterations in both the physical and technical parameters associated with soccer kicking performance and encourages players to shoot into more complex zones. Additionally, da Silva, [47] demonstrated that a differential learning intervention can improve the performance of pass technique in soccer and futsal players. Supporting these findings, Schollhorn et al., [48] affirmed that two techniques can be easily learned in one training session by applying the DL approach. Poureghbali et al., [49] also affirmed that applying DL inspires players to discover and explore more since the environment is ideally suited for fostering exploration behavior and helps to avoid the monotonous flow of repetitive training. Furthermore, Ozuak & Çaglayan, [50] approved that DL enhances the capacity of ball-dribbling and leads to flexible movement solutions in soccer. DL induces improvements in all dimensions, especially in the creative thinking ability of soccer players [11]. Santos et al., [16] supported that a DL training program facilitates the development of creativity components, namely the adaptability of movements, and promotes a decrease in failures among youth soccer players. Moreover, the non-linear pedagogical approach of DL provides a flexible and adaptable learning environment that satisfies the unique needs and preferences of each participant [4]. Unlike traditional linear practice approaches that prescribe fixed drills and repetitions, DL encourages self-organization and self-regulation, allowing learners to explore movement solutions and discover optimal performance strategies [19].

In contrast, while SSGs have been widely recognized for their effectiveness in promoting decision-making, tactical understanding, and team cohesion in soccer [22], our findings suggest that they may not be as conducive to technical skill development compared to DL. This divergence may be attributed to the constrained and structured nature of SSGs, which may limit the variety of movement patterns and solutions explored by participants [14]. Many researchers have explored the impact of SSGs on both physiological and technical demands, and they have affirmed that employing this approach primarily affects physiological rather than technical demands [51]. Their results concluded that SSGs had a more noticeable effect on physiological responses, such as heart rates, than on technical performance metrics. This emphasize the notion that while SSGs may elicit increased physical exertion and cardiovascular demand, they may not necessarily lead to significant improvements in technical skills [52–55]. The results of Casamichana & Castellano [56] suggest that while SSGs may influence physiological and performancerelated aspects, they may not be as effective in promoting certain motor behaviors and technical skills in soccer like interception, control and dribble, control and shoot, clearance, and putting the ball in play. In their study, Owen et al. [54] indicated that the variation of the field dimensions during SSG games did not impact the technical actions executed by players.

In the same context, some studies resulted in a higher frequency of duels and fewer technical actions, including successful passes and instances of ball loss [57–59]. Another study suggested that the reduction in the number of technical actions observed during SSGs could be attributed to psychophysiological stress experienced during these games [60]. A study conducted by Clemente et al. [31], investigating the acute effects of SSGs on physiological, physical, and technical performance in soccer, found that some forms of SSGs don’t have a significant impact in order to enhance technical performance. Furthermore, the fatigue induced by SSGs could have a direct effect on the work rate of individual players, thereby influencing their engagement in the game. This could potentially result in a decrease in the frequency of technical actions performed and the duration for which the ball remains in play [23, 58, 61]. These findings suggest that the physiological demands of SSGs may impact players’ ability to execute technical skills effectively during the game. In addition, the competitive and outcome-oriented nature of SSGs may shift the focus away from skill acquisition towards winning or achieving immediate performance outcomes.

### Limitations

While we tried to quantify technical skill enhancements, some measures might still be prone to observer bias, especially in the evaluation of tactical behaviors and decision-making skills. Despite our results demonstrating the effectiveness of SSG + DL, several potential confounding factors and limitations should be acknowledged.

Environmental conditions during the training sessions, though controlled between groups, may have influenced performance outcomes. Despite conducting sessions at similar times of day (17:00–18:30) with temperatures between 20–25°C, natural fluctuations in weather conditions could have affected physiological responses and performance metrics. The controlled training environment may not entirely reflect the complexities and pressures of actual competitive scenarios, potentially limiting ecological validity.

Individual differences among participants presented another limitation. Players with higher intrinsic motivation or superior baseline fitness levels might have responded more favorably to either training approach, potentially influencing the magnitude of improvements observed [62]. The psychological state of the players, including factors such as fatigue, stress, and emotional well-being, could have impacted their receptiveness to learning and skill acquisition during the intervention period. Furthermore, the SSGs and DL interventions were designed for specific technical skills, potentially overlooking other aspects of soccer performance.

Although we monitored physiological responses, the study did not adequately consider individual variations in fitness levels or external factors such as diet and rest, which could affect these measures and technical performance. Additionally, the study did not address the potential risk of overtraining or injury due to the frequency and intensity of the sessions, which is crucial for participant safety and well-being [63].

Future research should address these limitations through larger sample sizes to enhance statistical power and improve generalizability across diverse soccer populations. Studies exploring the effects of combined SSG + DL training across different age categories and skill levels would provide valuable insights into the developmental appropriateness of these methods. Longitudinal designs examining retention of acquired skills over extended periods would better assess the long-term efficacy of these training approaches.

## CONCLUSIONS

This study demonstrated that adding DL to SSGs did not increase cardiovascular or perceptual strain. Both groups experienced similar moderate rises in maximal heart rate and RPE, with no significant differences in heart rate, RPE, or blood lactate responses between SSG + DL and SSG alone. In contrast, tactical performance was markedly enhanced by DL when the SSG + DL group improved decision-making (8.84% vs. 5.64%), boosted successful tackles (68.41% vs. -1.22%) and increased possession maintenance (19.64% vs. 8.75%) in the SSG group. Technical skill execution also benefited more from the combined approach, with successful passing climbing 26.25% vs. 11.46%, alongside large effect sizes across shots and dribbles. Together, these findings demonstrate that integrating DL into SSGs accelerates tactical and technical development without placing additional physiological load on players.

From a practical perspective, these findings have considerable implications for coaches working with youth and amateur teams. The integration of DL components into small-sided games offers a cost-effective training strategy that maximizes skill development without requiring additional resources or training time. This approach may be particularly valuable for teams with limited facilities or coaching support, as it optimizes the learning potential within standard training sessions.

Future research should examine the long-term retention of skills developed through combined SSG + DL training and investigate its applicability across different competitive levels. Additionally, studies exploring the transfer of training effects to actual match performance would further validate this approach. Research into the optimal progression of DL elements within training cycles and their potential applications for injury prevention and rehabilitation would also be valuable extensions of this work.

## Declaration

In order to correct and improve the academic writing of our paper, we have used the language model ChatGPT-4o [64, 65]. After using this tool, the authors have reviewed and edited the content as necessary and take full responsibility for the content of the publication.
